# Design and evaluation of four low-cost engineering controls for reducing particle emissions from 3D printing

**DOI:** 10.1016/j.aeaoa.2026.100432

**Published:** 2026-04

**Authors:** Colleen O’Connor, Christopher Barnes, Kevin L. Dunn, Duane Hammond

**Affiliations:** Division of Field Studies and Engineering, National Institute for Occupational Safety and Health, Centers for Disease Control and Prevention, Cincinnati, OH, 45226, USA

## Abstract

Multiple studies indicate that workers are being exposed to emissions from material extrusion three-dimensional (3D) printing processes and suffering adverse health effects from the exposures. To mitigate these exposures, we developed and evaluated four low-cost engineering controls to capture particle emissions during 3D printing processes. Two controls (Controls A and B) were custom developed using 3D printed parts and retrofitted onto the extruder of two open-frame desktop fused filament fabrication (FFF) 3D printers (Printers 1 and 2) to capture emissions near the source. A third control (Control C) used a commercially available, printer-specific enclosure that partially encapsulated Printer 2, while a fourth control (Control D) was built using acrylic panels to fully enclose Printer 2. Particle emissions from each control were exhausted through NIOSH-designed air filtration units consisting of a high-efficiency filter, blower fan, and 3D printed housing/components. Control effectiveness was evaluated in an emissions test chamber by comparing average particle concentrations at the chamber outlet while 3D-printing a National Institute of Standards and Technology (NIST) test artifact with black acrylonitrile butadiene styrene filament, with and without the controls in operation. Minimum capture efficiencies were 99.7% for Control A, 91.8% for Control B, 28.6% for Control C, and 99.8% for Control D. Internal cooling fans in Printer 2 allowed particle emissions to bypass Control C, limiting its effectiveness. Overall, capturing emissions at the source (Controls A and B) and fully enclosing the printer (Control D) were highly effective and demonstrated that low-cost engineering controls can mitigate particle emissions from 3D printing processes.

## 1. Introduction

Three-dimensional (3D) printing is a popular way to create 3D objects that is rapidly moving into mainstream manufacturing. In 2021, more than 2.2 million 3D printers were sold, and shipments are expected to reach 15.3 million units by 2028 (Grand View Research, 2021). Fused filament fabrication (FFF), a type of material extrusion process, is the most common 3D printing technology because it is generally the easiest to access and operate and has lower initial equipment costs compared to other 3D printing technologies. During the FFF 3D printing process, thermoplastic material held in a spool is fed into a heated nozzle where it is melted and then deposited onto a build plate. This process is repeated, layer upon layer, until a solid object is formed. FFF 3D printers can produce models ranging from prototypes and test components to end-use parts depending on the application and material used. However, 3D printing is still a relatively new and rapidly growing industry, and studies continue to indicate that workers are being exposed to emissions from 3D printing processes (Geiss et al., 2016; Gu et al., 2019; Väisänen et al., 2018; Zontek et al., 2017) and experiencing health effects such as asthma or allergic rhinitis from the exposures (Chan et al., 2018; House et al., 2017; Stefaniak et al., 2017a). Emissions released during FFF 3D printing include volatile organic compounds and particulates, including ultrafine particles (UFPs; particles less than 100 nm in size) which are produced from the melted material extruding from the nozzle. However, these emissions released from 3D printing processes can be reduced by using engineering controls.

Engineering controls reduce or prevent hazards from coming into contact with workers and may include modifying equipment or workspace, using protective barriers, ventilation, and more. According to the National Institutes for Occupational Safety and Health (NIOSH) the most effective engineering controls are (1) part of the original equipment design, (2) remove or block the hazard at the source before it comes into contact with the worker, (3) prevents users from modifying or interfering with the control, (4) need minimal user action for the controls to work, and (5) operate correctly without interfering with the work process or making the work process more difficult (NIOSH, 2024). A review by Du Plessis et al. (2022) identified 29 published studies that have implemented engineering controls to mitigate 3D printer emissions across various types of technologies, including 20 studies focused on FFF printers. Multiple studies included in this review focused on evaluating the effectiveness of general ventilation, enclosures, local exhaust ventilation, and combinations of these control solutions for reducing emissions from various 3D printing processes (Väisänen et al., 2018; NIOSH, 2017; Kwon et al., 2017; Secondo et al., 2020; Dunn et al., 2020; Viitanen et al., 2021; Zontek et al., 2021).

Viitanen et al. (2021) found that general ventilation is not a sufficient exposure control for 3D printing emissions and that a canopy hood above a 3D printer was also ineffective; however, they found that a retrofitted ventilated enclosure exhausting to outdoor air reduced 3D printing emissions by 96% and a retrofitted non-ventilated enclosure reduced emissions by 89%. Kwon et al. (2017) evaluated eight different combinations of control methods used to reduce particle emissions during 3D printing and found that a ventilated enclosure with a high-efficiency particulate air (HEPA) filter removed 99.95% of nanoparticles. Researchers from the National Institute for Occupational Safety and Health (NIOSH) found that a custom-built ventilated enclosure effectively reduced particle concentrations in a 3D printing room by over 99% and reduced the total organic chemical concentration by almost 70% (NIOSH, 2017). NIOSH researchers also published a study evaluating a low-cost, easy to use local exhaust ventilation (LEV) engineering control that attaches directly to the extruder of a common desktop 3D printer capable of reducing particle emissions by around 98% in laboratory conditions. The same researchers also evaluated this control in a simulated makerspace with 20 printers operating simultaneously, where particle concentrations were around 20,000 particles/cm^3^ without the controls in operation and remained at or below background levels (<1000 particles/cm^3^) when the engineering controls were operating (Dunn et al., 2020). The engineering control evaluated in this study was published on the National Institutes of Health (NIH) 3D print exchange at Discover 3D Models | NIH 3D Print Exchange under model #: 3DPX-015467 and includes the assembly instructions and part files to make them free and available to download (National Institutes of Health). These studies show that low-cost engineering controls can be implemented into the FFF 3D printing process to reduce particle emissions.

This paper will discuss the design and evaluation of four additional examples of local exhaust ventilation controls for FFF 3D printers, supporting NIOSH’s broader effort to develop and assess affordable and practical engineering control solutions for a range 3D printing technology. The controls were evaluated under laboratory conditions and may not fully reflect real-world performance. Nevertheless, the controls represent two possible styles of custom engineering controls that can be designed and installed on individual open-frame desktop 3D printers to reduce worker exposure to emissions. Before retrofit engineering controls are added to 3D printers in the workplace, a qualified safety and health professional should conduct a risk assessment to ensure that the addition of the engineering control does not present an increased risk of fire or other hazards. Additionally, it should be determined if incorporation of the control will void any manufacturer warranties or certifications. This will allow the printer owner to make an educated decision on the risk versus reward of adding the control before proceeding. Engineering controls introduced by the manufacturer into the original design of a 3D printer are preferred, over retrofit controls. However, studies that show effectiveness of retrofit engineering controls may also be useful for manufacturers of 3D printers as more companies work toward introducing engineering controls as optional or standard features on 3D printers.

## 2. Methodology

### 2.1. Development of four engineering controls

We designed local exhaust ventilation (LEV) controls for two commercially available desktop 3D printers, Printer 1 and Printer 2. Detailed specifications for Printers 1 and 2 are summarized in Table S1 in the Supplemental Information (SI) file. Table 1 details all LEV controls evaluated on Printers 1 and 2 described below. Fig. 1 illustrates how the controls were operated during experiments. The control for Printer 1, referred to as Control A, was designed to attach easily to the printer’s extruder without the need for tools or disturbing normal operating practices, enabling capture of particle emissions near the source. The control can also be readily removed to perform maintenance of the extruder head. It was designed using a commercially available computer-aided design (CAD) software program, SOLIDWORKS^®^ (Version, 2021; Dassault Systemes, Velizy-Villacoublay, France) (Fig. 2) then 3D printed and attached to Printer 1 (Fig. 3). The part was designed and formatted to print on Printer A with acrylonitrile butadiene styrene (ABS) filament. The printed control was used in conjunction with parts from another NIOSH-designed 3D printer LEV control (Dunn et al., 2020), which included a H13 HEPA vacuum filter made with glass fiber media (Model 923480–01, Dyson Inc., Chicago, IL, USA); a single-speed 12VDC radial blower (Model JT-FS-0002-1232-12, UTUO, Shenzhen, China); flexible CPAP tubing with 22 mm cuffs (Model B01MU5XLUC, RespLabs Medical Inc., Ferndale, WA, USA); and 3D-printed housing to contain the HEPA filter and an inlet to connect Control A to the LEV system with the tubing (Fig. 4).

The second 3D printer, Printer 2, was evaluated with three separate controls, Control B, Control C, and Control D. Controls B and C were designed and fabricated using the same CAD-based workflow, tool-free attachment, and removability features described for Control A, with control-specific modifications described below. Control B was 3D printed using ABS filament on Printer 2 (Figs. 5 and 6). It attaches directly to the printer’s extruder without tools and connects to the same HEPA filtration unit used for Control A for Printer 1.

Control C is a commercially available polycarbonate enclosure available from e-commerce retailers designed specifically to encapsulate the volume surrounding Printer 2’s build plate while excluding the cooling fan for the printer’s electrical components (Fig. 7). This enclosure includes panels that were designed to fit into the openings on each side of the 3D printer, along with larger inserts that enclose the build platform and includes a door to allow access to the built parts and to facilitate maintenance activities. The top panel includes a slot to accommodate the filament holder, feeding filament to the extruder head, and movement of the extruder head. Assembling the enclosure does not require any tools, as all pieces either snap together or fit snuggly into the openings of the 3D printer. A custom exhaust duct was designed by NIOSH engineers using CAD software and 3D printed with ABS filament to attach directly to the small opening on the left side of the printer. The duct connects to the intake of a modified version of the HEPA filtration unit used with Controls A and B (Fig. 4), in which the outer diameter of the intake was increased from 2.5 cm to 5.5 cm to provide ventilation for the enclosure.

Control D was designed to fully enclose Printer 2 while remaining easily removable to allow access to printed parts and to service the printer as needed. It consists of a small enclosure assembled using common shop tools (e.g., CNC machine and hand tools). The assembly is comprised of clear acrylic panels glued together, with an acrylic lid with a foam gasket applied around its perimeter to form a seal with the top of the walls of the enclosure (Fig. 8). The enclosure measures 40.6 × 50.8 × 39.4 cm (16 × 20 × 15.5 in), with the lid measuring 41.9 × 52 cm (16.5 × 20.5 in). The lid additionally has a slot to accommodate the filament holder and allow filament feeding into the 3D printer’s extruder. A 23.5 × 5.2 cm (9.25 × 2.1 in) cutout was made near the bottom right side of the enclosure to provide makeup air (Fig. 3). On the left side of the enclosure, a 6.35 cm (2.5 in) diameter hole was cut near the top of the enclosure to accommodate 6.35 cm (2.5 in) flexible tubing, allowing the enclosure to connect to a filtration system for ventilation. This design approach was taken to accommodate the upward movement of particles driven by convective forces generated by heat from Printer 2’s extruder and build plate. The filtration system for Control D is similar to that shown in Fig. 3, with some modifications including a larger 3D printed filter housing designed to accommodate a 15.5 cm (6.1 in) diameter × 13 cm (5.1 in) thick five-layer pleated paper shopvac filter (Model VF3600, RIDGID^®^ Wet/Dry Vacs, St. Louis, MO, USA). The filter has a reported capture efficiency of 99% for particles ≥0.5 μm. Additionally, the outer diameter of the filter housing’s intake was increased from 2.5 cm to 6.3 cm to ensure a secure connection with the 6.35 cm tubing.

An exhaust airflow rate was measured through each engineering control by connecting to a mass flow meter (Model 620S-RFQ-4710, Sierra Instruments, Inc., Monterey, CA, USA). The airflow rate was 6.3 m^3^/h (3.7 cubic feet per minute [cfm]) for Control A, 2.4 m^3^/h (1.4 cfm) for Control B, 12.1 m^3^/h (7.1 cfm) for Control C, and 21.2 m^3^/h (12.5 cfm) for Control D.

### 2.2. Evaluation of four engineering controls

The effectiveness of Controls A – D was evaluated using an experimental setup adopted from Mendes et al. (2017). To conduct each control evaluation, the 3D printer (along with its corresponding LEV control, when appropriate) was placed inside a HEPA filtered emissions testing chamber located in a 3.81 × 3.82 × 2.46 m (12.5 × 12.54 × 8.07 ft) room. The room was supplied with HEPA filtered air in a crossflow pattern, with return air exhausted to outdoor air, to eliminate background particles. The emissions testing chamber is a 0.22 m^3^ (8 cubic-foot (ft^3^)) chamber constructed from 80/20 aluminum and acrylic sheets. The chamber inlet connects to a 20 cm (8-inch) diameter flexible duct in series with a portable air cleaner with HEPA filtration (Model SS-300-PYT, Sentry Air Systems, Inc., Houston, TX, USA) which provides constant air flow through the chamber (Figs. 9 and 10). The chamber outlet also has a 20 cm (8 in) diameter flexible duct connected on the opposite side from the portable air cleaner to measure chamber airflow rate and allow for collection of particle emissions during the test prints. Air flow rate was measured using a balometer (Model EBT731,TSI Inc., Shoreview, MN, USA). Average air flow was measured to be 60 cubic meters per hour (m^3^/hr) (35 cfm). Particle emissions were sampled real-time using a NanoScan Scanning Mobility Particle Sizer (SMPS) (Model 3910, TSI Inc., Shoreview, MN, USA) and a Condensation Particle Counter (CPC) (Model 3007, TSI Inc., Shoreview, MN, USA). Both instruments were used for data collection since each has different collection rates and size ranges. The NanoScan measures particles from 10 to 420 nm in size every minute allowing for a total number concentration up to 1,000,000 particles/cm^3^ and particle size distribution data in 13 size channels. The CPC measures number concentrations for a size range from 10 to ~1000 nm every second in a single size bin up to a maximum total number concentration of 100,000 particles/cm^3^. Size distribution data from the NanoScan for Printers 1 and 2 showed that nearly all measured particles were below 200 nm, with a peak particle size of 15.4 nm (Fig. S5). Total number concentration of particles measured per trial were very similar between instruments. Therefore, only results from the NanoScan were used for the performance analyses described in this report and the CPC data were used as a check to verify consistency with the readings from the NanoScan. Both instruments were factory calibrated by the manufacturer prior to data collection.

The effectiveness of each control was evaluated using randomized paired trials conducted with and without the control in operation. Ten successful tests pairs (twenty individual trials) were performed for each printer. All tests were conducted printing the National Institute of Standards and Technology (NIST) test artifact (Fig. 11), originally designed to assess the performance of 3D printing systems (Moylan et al., 2012). The NIST test artifact was scaled to take approximately 1 h to print for Printers 1 and 2. All test prints were done with Matterhackers Build Series brand black ABS filament (Matterhackers Inc., Lake Forest, CA, USA) with the extruder temperature set to 240 °C and bed temperature set to 100 °C for both Printers 1 and 2 across all tests.

### 2.3. Statistical procedures

Once all test prints were completed, both with and without Controls A – D attached and operating on Printers 1 or 2, the emissions data obtained from the NanoScan and CPC were analyzed using Microsoft Excel (Version Office 365, Microsoft Corporation, Redmond, WA, USA) to calculate the average particle concentration measured at the outlet of the chamber for each trial. Capture efficiency for each control was calculated by comparing average particle concentrations from randomized paired trials conducted with Controls A – D attached but not operating to those from trials in which the same controls were attached and operating on the same printer. These comparisons were performed separately for Printers 1 and 2 using Equation (1).

Equation (1): $$\eta =\frac{C_{\text{off}}-C_{\text{on}}}{C_{\text{off}}}\times 100$$

Where.

η = capture efficiency

*C*_*off*_ = average particle concentration measured at the outlet of the chamber during a trial when the engineering control was turned off

*C*_*on*_ = average particle concentration measured at the outlet of the chamber during a trial when the engineering control was turned on.

Minimum overall capture efficiency of each engineering control was calculated following the procedures described in NIOSH Publication No. 97-105 (NIOSH, 1997). Since all tests were conducted inside of a HEPA filtered environmental test chamber located within a HEPA filtered room, it was not necessary to correct for background particles. We measured a zero-particle concentration on both instruments before beginning 3D printing each trial. During 3D printing trials, number concentration of particles as a function of time plots were similar to another 3D printing particle emission study that used ABS filaments (Dunn et al., 2020).

We determined the minimum capture efficiency for each evaluated engineering control using Equation (2) to calculate the test statistic (T) which represents the lower 95% confidence level. The mean capture efficiency (m) was calculated by taking the average reduction in particle emissions across the randomized paired trials. The estimated standard deviation (s) was calculated for the set of n = 10 randomized paired trials. The value for (t) was selected from the Student’s t-distribution table at the 95th percentile based on the value of (n-1), i.e. (10–1).

Equation (2): $$T=m-\frac{\left(t-s\right)}{n^{0.5}}$$

## 3. Results

The minimum capture efficiency at the lower 95% confidence interval for Controls A – D ranged from 28.55 to 99.78%. Across all tests conducted with Control A – D, peak particle concentrations were typically seen around the 10-min mark for Printer 1 and the 5-min mark for Printer 2, followed by a rapid decline in particle concentrations. Control-specific capture efficiencies and deviations from these general temporal emissions trends are described in the results presented below. All calculations for capture efficiency and plots of particles emissions measured for each test are provided in the SI file.

The average particle concentrations with and without Control A attached and operating on Printer 1 while conducting experiments can be seen in Table 2, which shows a consistent reduction in particle concentrations. Without Control A operating, the average particle concentrations emitted from Printer 1 ranged from 331 to 4408 particles/cm^3^. When the LEV control was in place and operating, average particle concentrations ranged from 0.20 to 16.22 particles/cm^3^. The average capture efficiency of all 10 tests was 99.87%. Based on this data, it can be concluded that with 95% confidence the efficiency of Control A while operating on Printer 1 is greater than 99.72% under the described laboratory conditions. Fig. S1 illustrates the temporal behavior of particle emissions from Printer 1 during Test 8, in which Control A was the most effective in reducing particle emissions. The temporal emission profile for Control A generally remained consistent across tests. A notable exception was Test 6 with Control A not in operation, where average particle concentrations peaked approximately 27 min into the experiment.

Average particle concentrations with and without Control B attached and operating on Printer 2 can be seen in Table 3. Similar to Control A, Control B shows a consistent reduction in particle concentrations during the printing of the NIST test artifact. Without Control B operating, average particle concentrations ranged from 918 to 85,130 particles/cm^3^. When Control B was in operation, average particle concentrations ranged from 10.83 to 6502 particles/cm^3^. The capture efficiency for each test ranged from 84.34 to 99.75%, with an average reduction of 95.26%. With 95% confidence the efficiency of Control B while activated on Printer 2 was greater than 91.75% under the evaluated laboratory conditions. Fig. S2 illustrates the temporal behavior of particle emissions from Printer 2 during Test 3, in which Control B was most effective in reducing particle emissions. Similar to Control A, the temporal emission profile from Printer 2 generally remained the same with and without Control B in operation. Test 10 was an exception, where average particle emissions remained elevated throughout the experiment both with and without Control B in operation.

Table 4 displays the average particle concentrations measured with and without Control C, the commercially available enclosure with LEV, operating with Printer 2. These results show a wide fluctuation in reductions from test to test. The average particle concentration without the Control C activated ranged from 397 to 4061 particles/cm^3^. While Control C was activated, average particle concentrations ranged from 33.98 to 1815 particles/cm^3^. The capture efficiency for each test ranged from 36.91% to 94.43%, with an overall average reduction of 57.39%, the lowest of all controls evaluated. Based on this data, with 95% confidence the efficiency of Control C is at least 28.55% under the evaluated laboratory conditions. Fig. S3 illustrates the temporal behavior of particles emission from Printer 2 during Test 6, where Control C demonstrated the greatest reduction in particle emissions. The temporal emission profile from Printer 2 remained consistent across tests conducted with and without Control C in operation.

The results for Control D, the NIOSH-built enclosure with LEV installed on Printer 2, can be seen in Table 5 and shows a consistent reduction in particle emissions while printing the NIST test artifact. Without Control D operating, average particle concentrations ranged from 1828 to 9826 particles/cm^3^. With Control D operating, average particle concentrations ranged from 1.86 to 20.23 particles/cm^3^. The capture efficiency for each test ranged from 99.68% to 99.90%, the highest among all controls evaluated. With 95% confidence, the capture efficiency of Control D was 99.78% under the evaluated laboratory conditions. Fig. S4 displays the temporal behavior of particle emissions from Printer 2 during Test 2, in which Control D was the most effective at reducing particle emissions. Similar to Control C, the temporal emission profiles remained consistent across all tests conducted with and without the control in operation.

## 4. Discussion

During our study, we found substantial variability in the particle emissions during experiments. Multiple studies have shown this inherent variability is common within FFF 3D printing (Gu et al., 2019; Mendes et al., 2017; Azimi et al., 2016; Zhang et al., 2017; Stefaniak et al., 2017b). Average particle concentrations measured at the outlet of our testing chamber varied greatly between and within the two models of 3D printers used in this study. The same print and bed temperature, brand and color of filament material, 3D print object (NIST test artifact), and chamber air flow rate were all held consistent throughout the study. However, we observed substantial trail to trail variability in measured particle concentrations during the study, with emission peaks occurring in the early stages of printing, following a trend reported in previous studies (Kwon et al., 2017; Mendes et al., 2017; Azimi et al., 2016; Kim et al., 2015). Despite this variability, Controls A, B, and D maintained a minimum capture efficiency ≥91%.

The final print (Test 10) for Control B installed on Printer 2 had a noticeably higher average particle concentration both with and without the control in operation. A failed print occurred prior to this final test set, in which the part did not print correctly, and the control became clogged with flakes of the extruded ABS filament. The control was removed from the extruder head and cleaned to ensure no remaining filament clogged the control. All subsequent tests, regardless of control usage, had elevated particle concentrations. It was unclear the exact reason for this increase in particle emissions, as the NIST test artifact continued to print correctly and there were no other obvious issues with the extruder or any part of the 3D printer. Despite this elevation in particles, the control still showed a 92.36% capture efficiency, better than two other test sets (Tests 5 and 8) that occurred prior to the failed print.

A similar deviation was observed in Test 6 for Control A operating on Printer 1, where the peak particle concentration shifted from the typical 10-min trend to approximately 27 min into the print. As with Control B, the cause of the shift could not be determined; however, it did not adversely affect control performance, as Control A still achieved a capture efficiency exceeding 99%. Additionally, several tests conducted with Controls A (Tests 4, 7, 8, and 10) and D (Tests 3, 6, and 9) in operation experienced increased sensitivity to transient particle concentration peaks when plotted. We attributed this behavior to the high performance of both controls, as Control A and B achieved the highest minimum capture efficiencies at the 95% lower confidence interval, and lowest observed average particle concentrations across all tests performed with a control in operation (2.17 particles/cm^3^ for Control A and 6.62 particles/cm^3^ for Control B). Furthermore, these tests consistently achieved capture efficiencies greater than 99%, aligning with the overall performance trends observed for both controls.

In contrast, during the evaluation of Control C, increased fluctuation of particle emissions from Printer 2 was observed between tests regardless of whether the control’s LEV was in operation. These fluctuations were much greater than the variability of 3D printing particle emissions observed with the other controls evaluated in our study. We noted that Printer 2 has a cooling fan and corresponding perforations through its housing to ventilate and cool the printer’s electronic components, which are not encapsulated by Control C. During printing this cooling fan exhausts particle-laden air through these perforations, bypassing Control C’s HEPA filtered LEV unit designed to capture particles emitted during the 3D printing process. Although Control C was ineffective, this set of tests demonstrated the importance of understanding the design of each 3D printer to ensure that selected engineering control solutions effectively capture particles emitted during 3D printing processes. Instead of designing a separate filtration system for the cooling fan with Control C, we designed and built a new enclosure (Control D) to fully enclose the 3D printer. Control D was much more effective compared to Control C, achieving a minimum capture efficiency of 99.78% compared to 28.55% at the 95% confidence interval, a difference of 71.19%. These findings reinforce the critical role of appropriate engineering control design and implementation in mitigating particle emissions from FFF 3D printers.

Control A and Control D were the most effective solutions we developed, achieving minimum capture efficiencies of 99.72% and 99.78%, respectively, with 95% confidence. An assessment of the design features indicated that Control A had a higher exhaust airflow due to the differences in airflow restrictions from the attachments that connect the hose to the 3D printer’s extruder. The higher exhaust airflow of Control A (6.3 m^3^/h [3.7 cfm]) allowed for a more efficient capture of particles during the printing process versus the lower flowrate of Control B (2.4 m^3^/h [1.4 cfm]), despite a similar strategy of capturing particles near the source. Although both Control A and Control B, designed for Printers 1 and 2, respectively, captured particles near the source, the geometry of Printer 2’s extruder limited clearance for positioning the hose inlet of the LEV system to be close enough to the nozzle to effectively capture particles. As a result, it was necessary to design the attachment for Control B with air inlet slots near the nozzle with narrow internal air passages within the 3D printed attachment to capture particles near the source. Routing air through Control B resulted in a lower exhaust airflow rate due to additional static air pressure losses, which resulted in a lower capture efficiency of particle emissions. Dimensional constraints around the extruder would not allow the attachment to be designed with larger air slots to reduce airflow resistance. Since all controls used a single-speed radial blower, the fan would have needed to be replaced with a more powerful model to increase the exhaust airflow rate for Control B, which may have increased capture efficiency. By contrast, Control D fully enclosed Printer 2, and with its filtration unit attached to the enclosure and operating, it was able to provide negative air pressure within the enclosure to effectively contain and remove the particle emission being produced during printing.

Additionally, print failures occurred during a couple of trials evaluating controls using Printer 2, which appeared to contribute to associated variabilities in particle count generation. These observations highlight the importance of inspecting and cleaning engineering controls following any print failures. This can prevent accumulation of debris which can lead to clogs that may restrict or block airflow and subsequently reduce the performance of the engineering control in use.

## 5. Limitations

We acknowledge that this study did have limitations. First, all controls were evaluated in laboratory conditions. The effectiveness of the controls evaluated may not directly translate into field settings, where variables such as user workflows, room ventilation, print temperature, variability in particle emissions from different filament manufacturers and types may impact performance. Follow-on studies evaluating the controls in real work environments would be beneficial to determine their effectiveness, impact on printer performance, and practicality with 3D printer workflows and maintenance activities in these settings. However, it is important to note that Dunn et al. (2020) evaluated an engineering control, designed similarly to Controls A and B, in a simulated 3D printer makerspace, where average particle concentrations reach 20,000 particles/cm^3^ without controls in operation and remained at or below background levels (<1000 particles/cm^3^) with the controls were operating. Additionally, NIOSH (2017) evaluated the effectiveness of a ventilated enclosure, similar in concept to Controls C and D, in a 3D printer room under field conditions and achieved a 99% reduction in particle emissions and near a 70% reduction in total volatile organic compounds. These findings demonstrate controls designed to capture emissions near the source or through a ventilated enclosure can be viable options in practical settings.

Next, this study focused on reducing particle emissions from 3D printing processes only. Individual volatile organic compounds (VOCs) and total volatile organic compounds (TVOC) were not sampled, but we acknowledge they can be released during 3D printing processes. The controls evaluated in this study were not designed to mitigate these compounds. However, if VOCs are the primary contaminants of concern, NIOSH (2023) provides recommendations for reducing both particle and gaseous emissions in their *Approaches to Safe 3D Printing: A Guide for Makerspace Users, Schools, Libraries, and Small Businesses* document, which is publicly available. The *2019 ASHRAE Handbook* also offers additional guidance on mitigating gaseous emissions, which includes VOCs (ASHRAE, 2019).

Finally, we did not investigate the detailed aerosol dynamics associated with particle emissions measured when evaluating engineering controls on Printers 1 and 2. Published studies evaluating particulate and gaseous emissions (e.g., VOCs) from fused filament fabrication 3D printers have demonstrated that they can undergo rapid, complex transformation involving nucleation, condensation, and coagulation when thermoplastic materials are heated and extruded from the printer nozzle (Zhang et al., 2018; Ding et al., 2019). However, we focused on evaluating the effectiveness of the controls by quantifying reductions in average particle number concentrations, rather than characterizing aerosol formation mechanisms.

## 6. Conclusions and recommendations

3D printing is an emerging technology commonly used in traditional workplace settings, such as manufacturing plants, academic institutions, and office environments. 3D printing is also used by self-employed/freelance workers and consumers for personal applications. In this study, NIOSH researchers developed four custom low-cost, retrofitted engineering controls (Controls A – D) that can easily be installed on two different fused filament fabrication (FFF) 3D printer models (Printers 1 and 2). A series of 10 randomized paired trials with and without engineering controls operating were performed for both 3D printers to assess the average particle concentrations, and to analyze the effectiveness of the NIOSH-designed engineering control attached to the 3D printers.

With 95% confidence, our study found that the NIOSH-designed custom engineering controls were able to reduce average particle concentrations by 99.72% for Control A when operated on Printer 1, 91.75% for Control B when operated on Printer 2, and 99.78% for Control D when operated on Printer 2. In contrast, Control C, which was purchased from an e-commerce retailer and then retrofitted with a HEPA filtered LEV system, was the least effective and consistent of all controls evaluated, with a minimum capture efficiency of 28.55% for particle emissions, with 95% confidence. These results emphasize the importance of understanding the design of each 3D printer to ensure the prospective engineering control solution will effectively and consistently mitigate target emissions during 3D printing processes. Additionally, the results from this study reflect performance of the evaluated controls in a controlled laboratory environment. Follow-on studies should be performed in both traditional and non-traditional as-used work environments to evaluate control effectiveness in practical settings. Factors such as room ventilation, room size, workflow, and 3D printer location should be considered and evaluated in these studies.

## Supplementary Material

Supplementary Material

## Figures and Tables

**Fig. 1.**
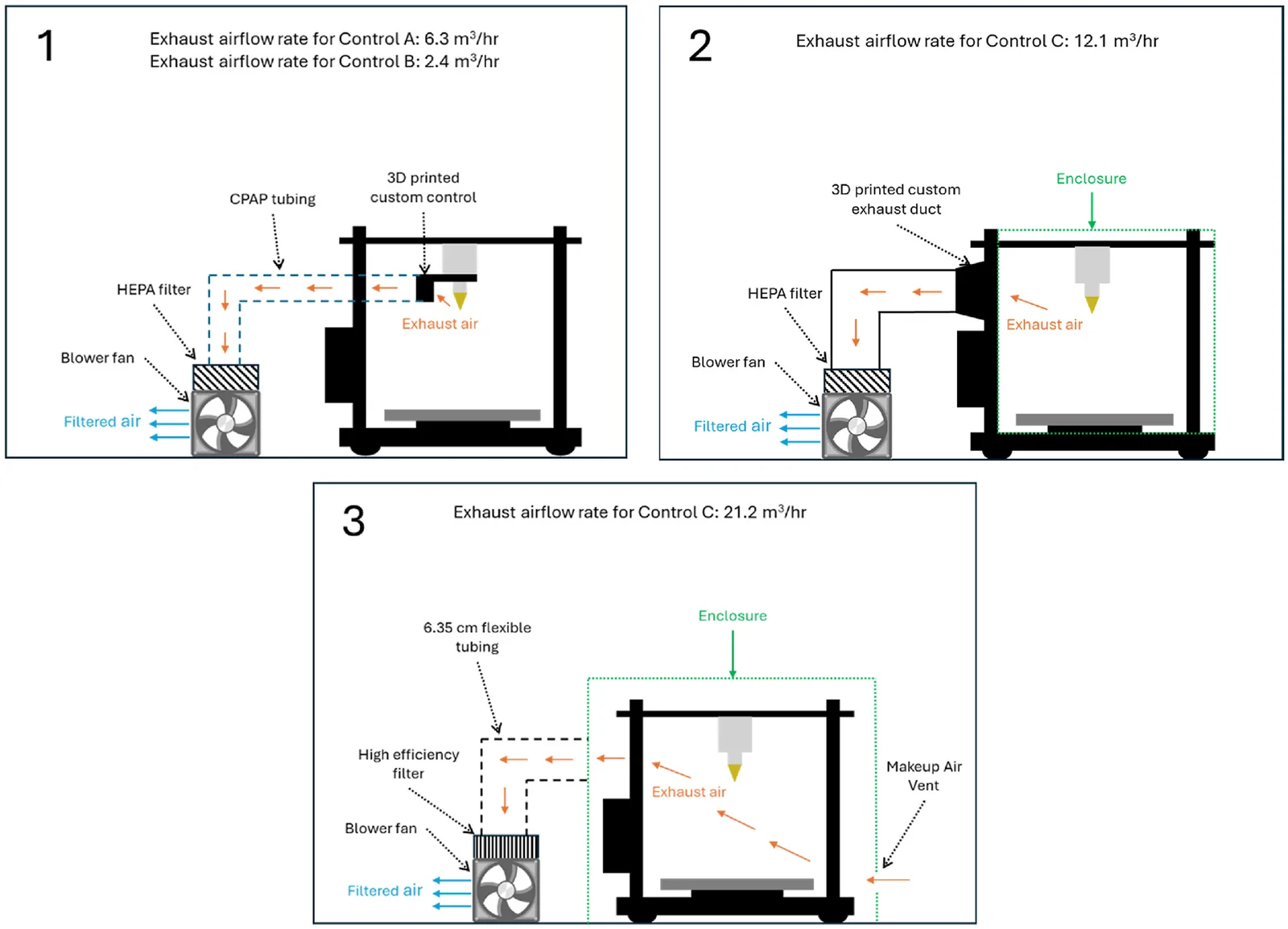
Operational setup of low-cost custom engineering controls (1) Controls A and B (2), Control C, and (3) Control D. Control A was evaluated on Printer 1, while Controls B – D were evaluated with Printer 2. Diagram by NIOSH.

**Fig. 2.**
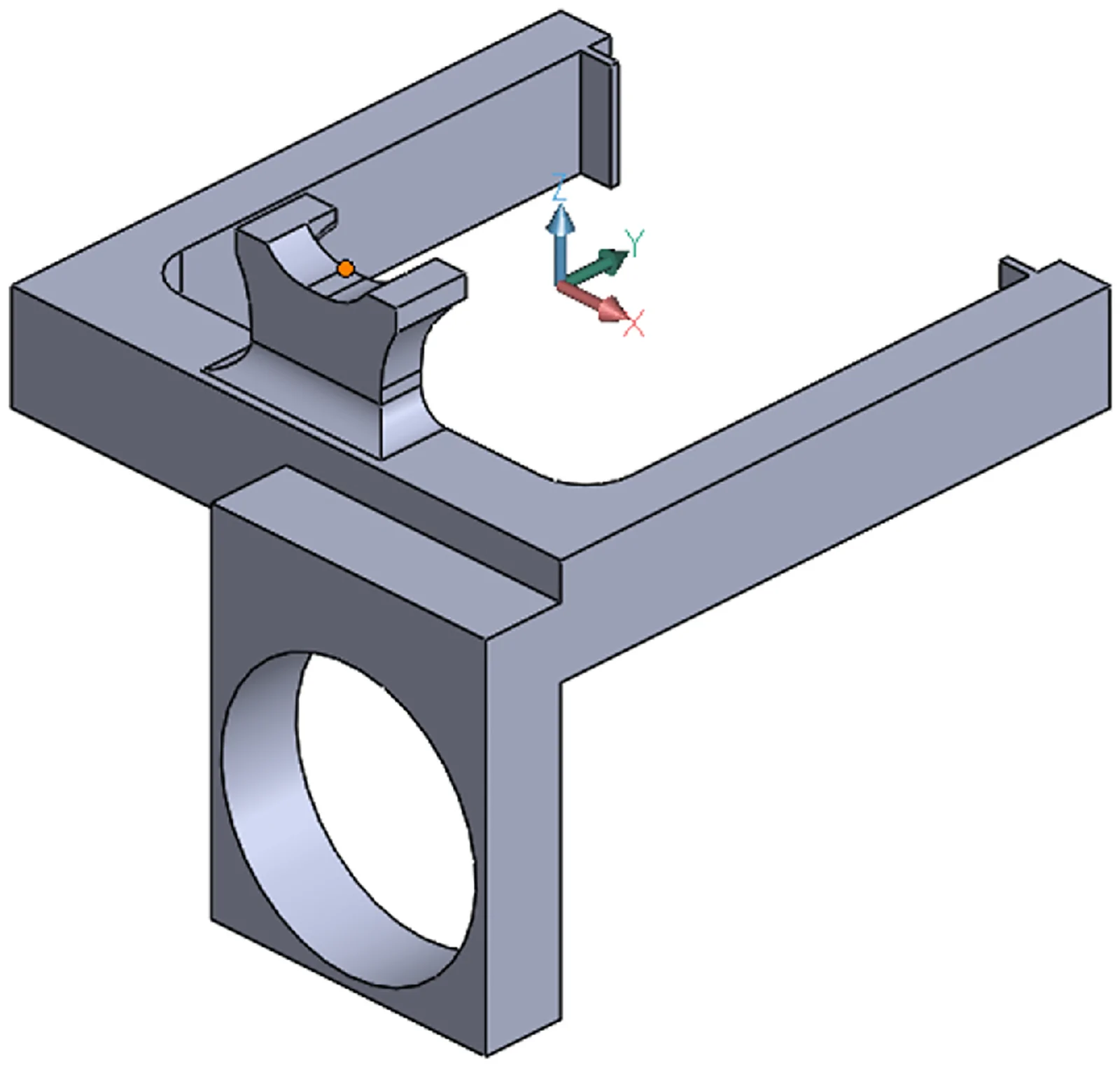
Localized engineering control attachment for Control A, designed to attach to the extruder head and remove emissions from the point of origin for desktop 3D Printer 1. Design drawing by NIOSH.

**Fig. 3.**
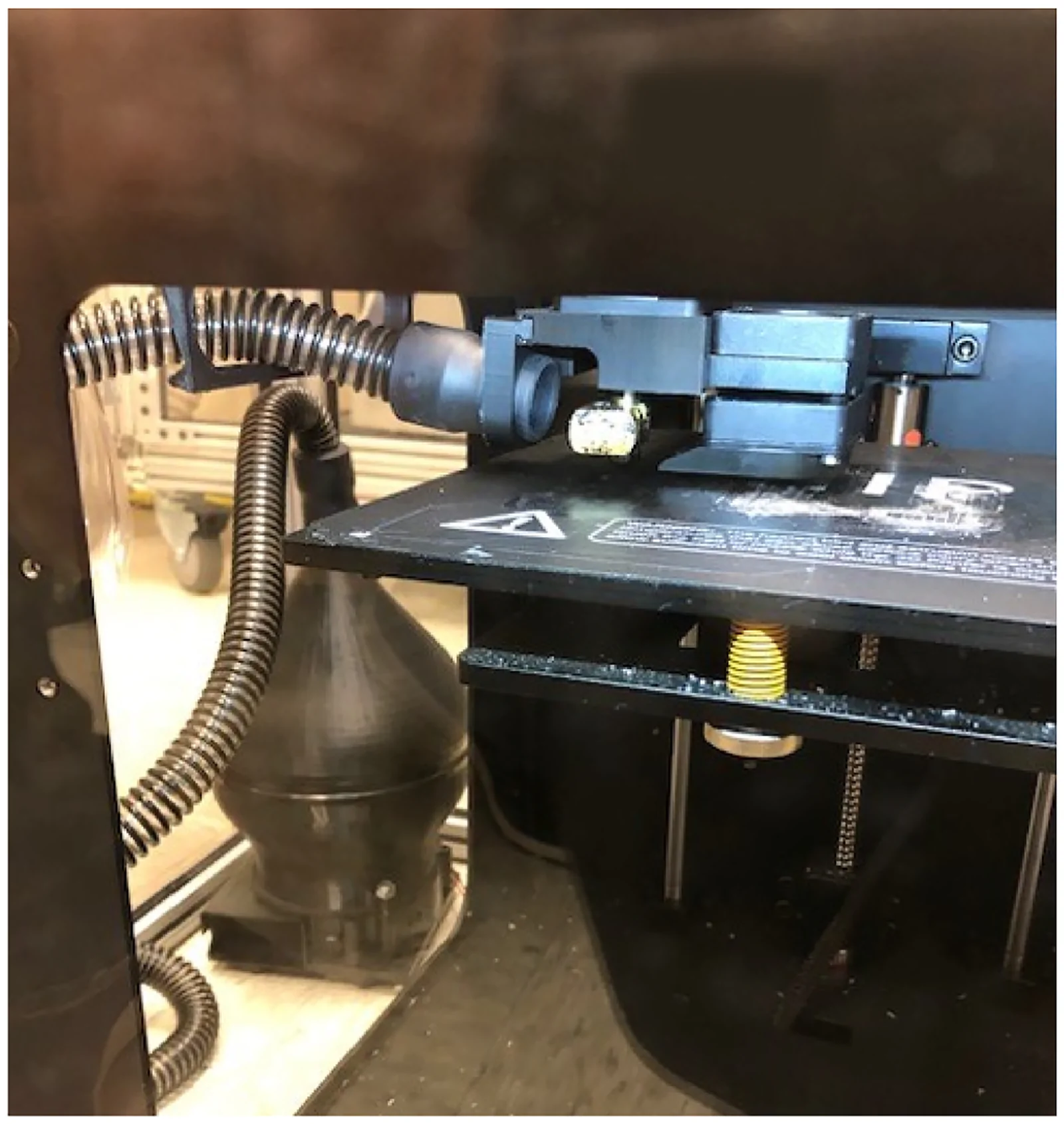
3D Printer 1 with LEV engineering control, Control A, attached. Photo by NIOSH.

**Fig. 4.**
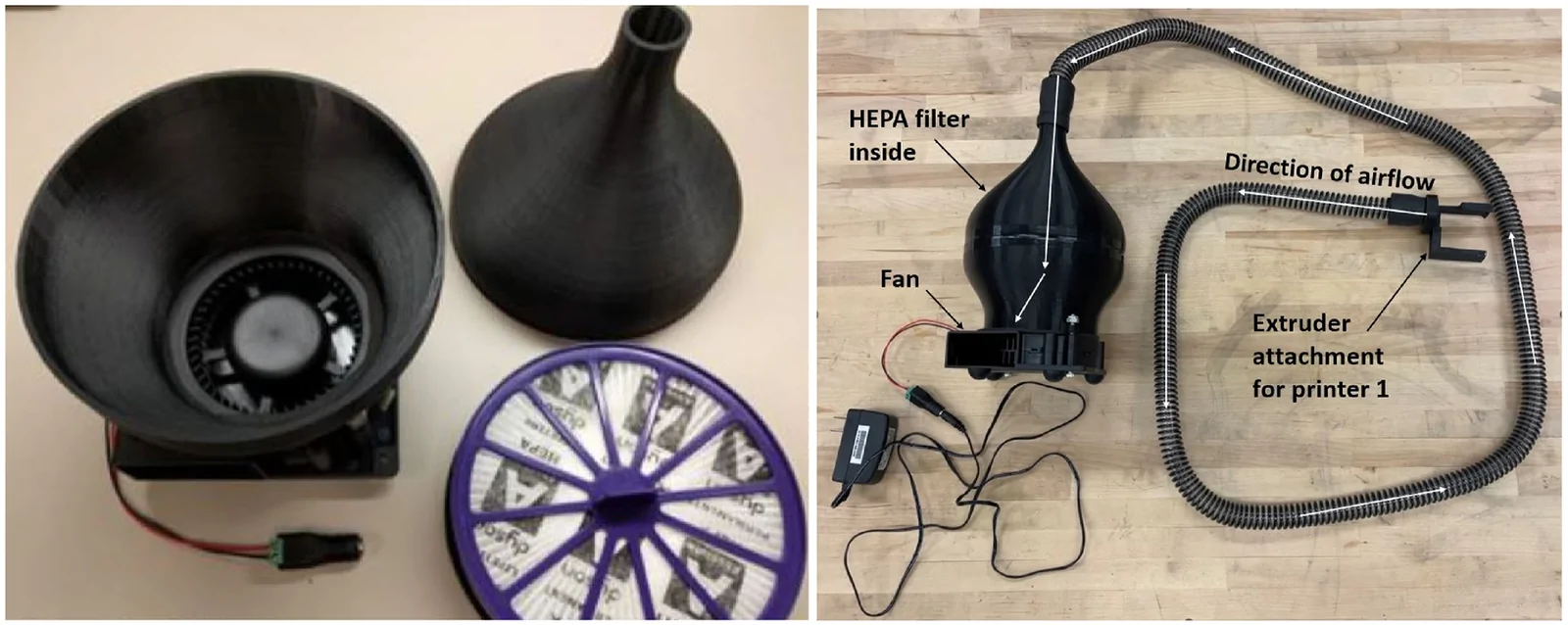
Low-cost air cleaner assembly connected to a 3D printer ventilation control piece. The HEPA filter is 14 cm (5.5 in) in diameter and 2.5 cm (1 in) in thickness. Hose is shown with Control A attached.

**Fig. 5.**
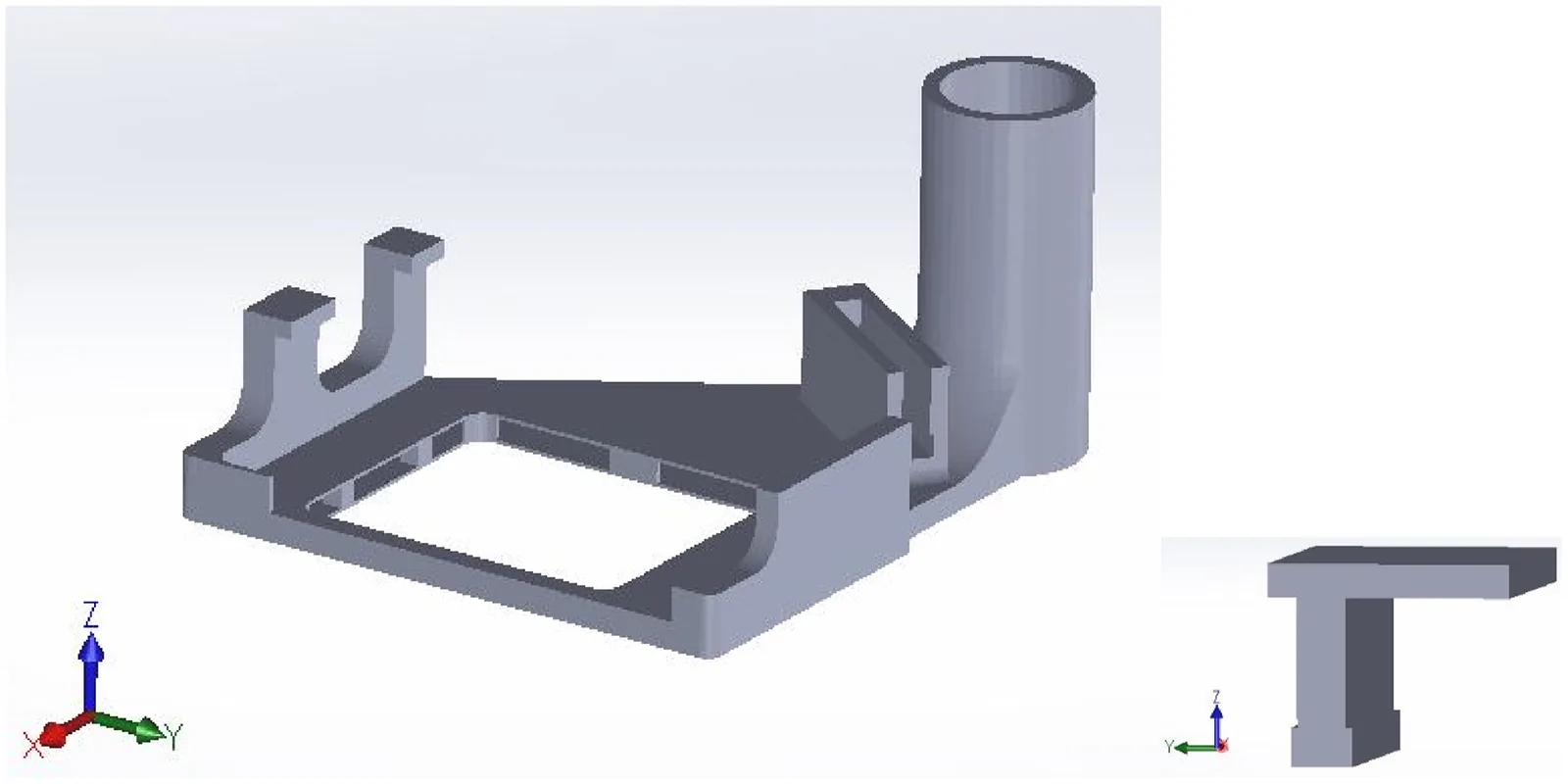
Control B LEV designed to remove emissions from the point of origin for desktop 3D Printer 2. Left is the main body of the control; right is the tab to secure the part to the extruder head. Design by NIOSH.

**Fig. 6.**
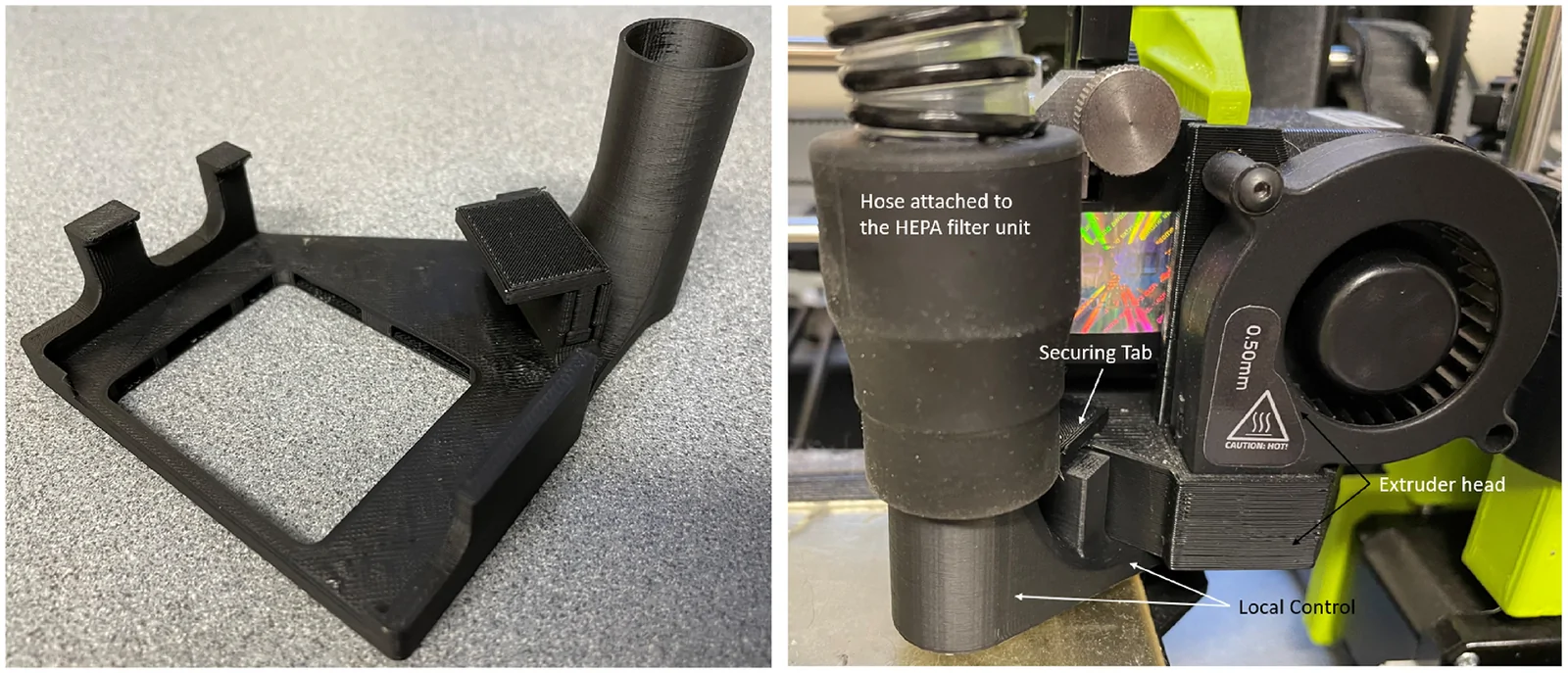
Printer 2 LEV Control B with tab inserted (left) and control attached to printer extruder head (right). Photos by NIOSH.

**Fig. 7.**
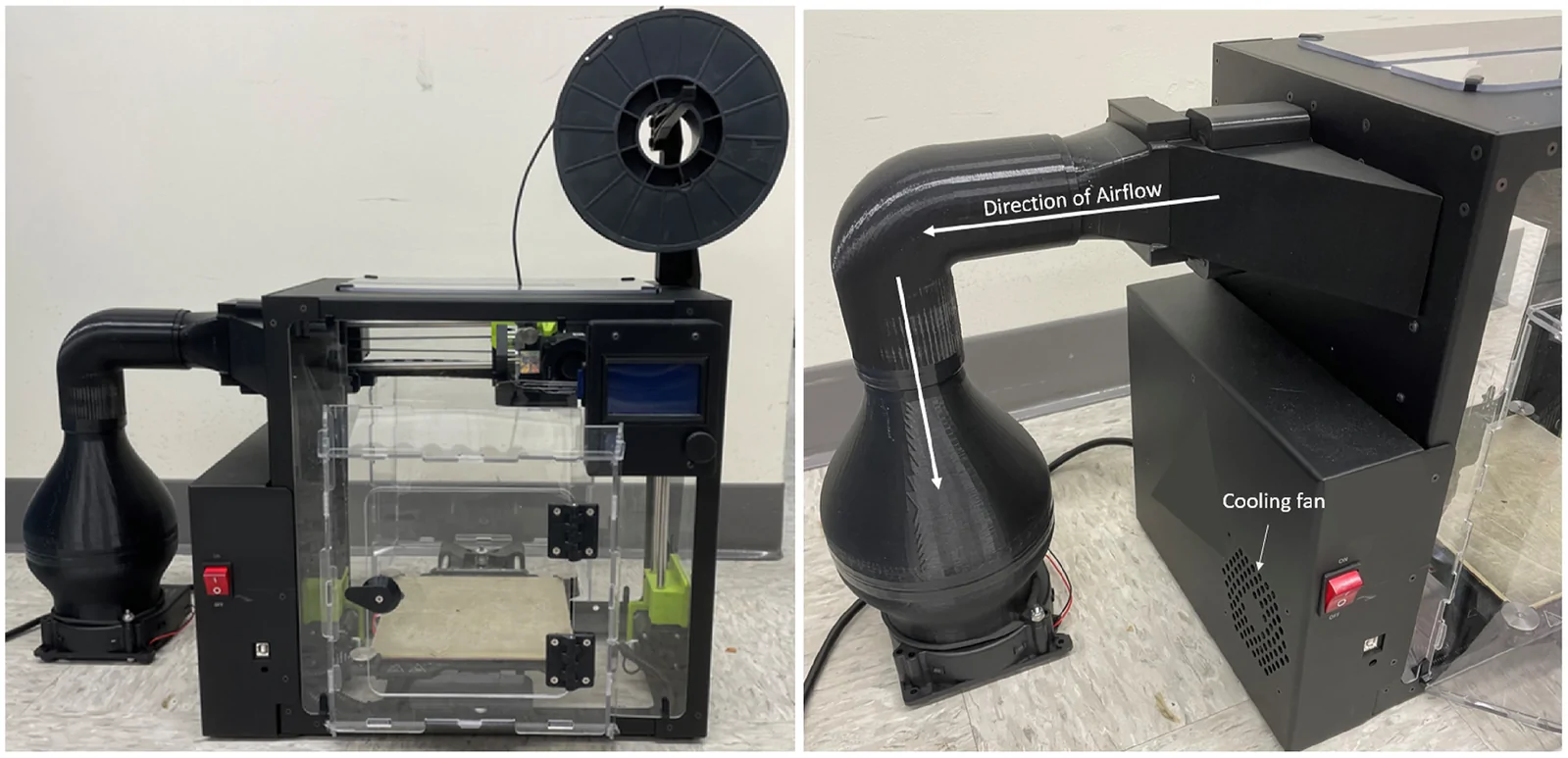
Printer 2 outfitted with commercially available enclosure and HEPA filtration unit. Image on right shows the cooling fan vent linked with particle reduction issues discussed below. Photos by NIOSH.

**Fig. 8.**
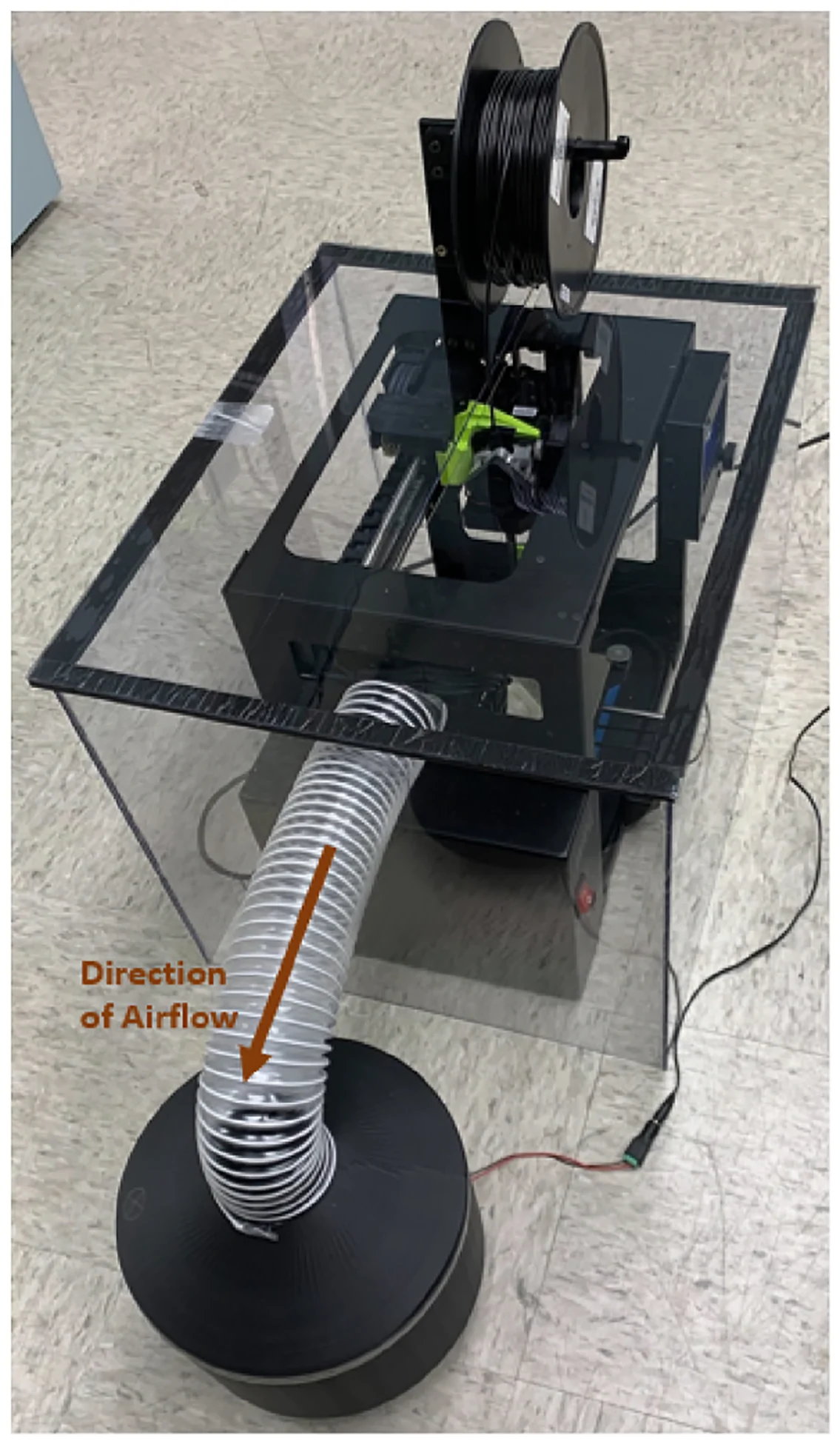
Printer 2 with Control D, the NIOSH-made enclosure with shopvac filtration unit attached. The filter is 15.5 cm (6.1 in) in diameter and 13 cm (5.1 in) in thickness Photo by NIOSH.

**Fig. 9.**
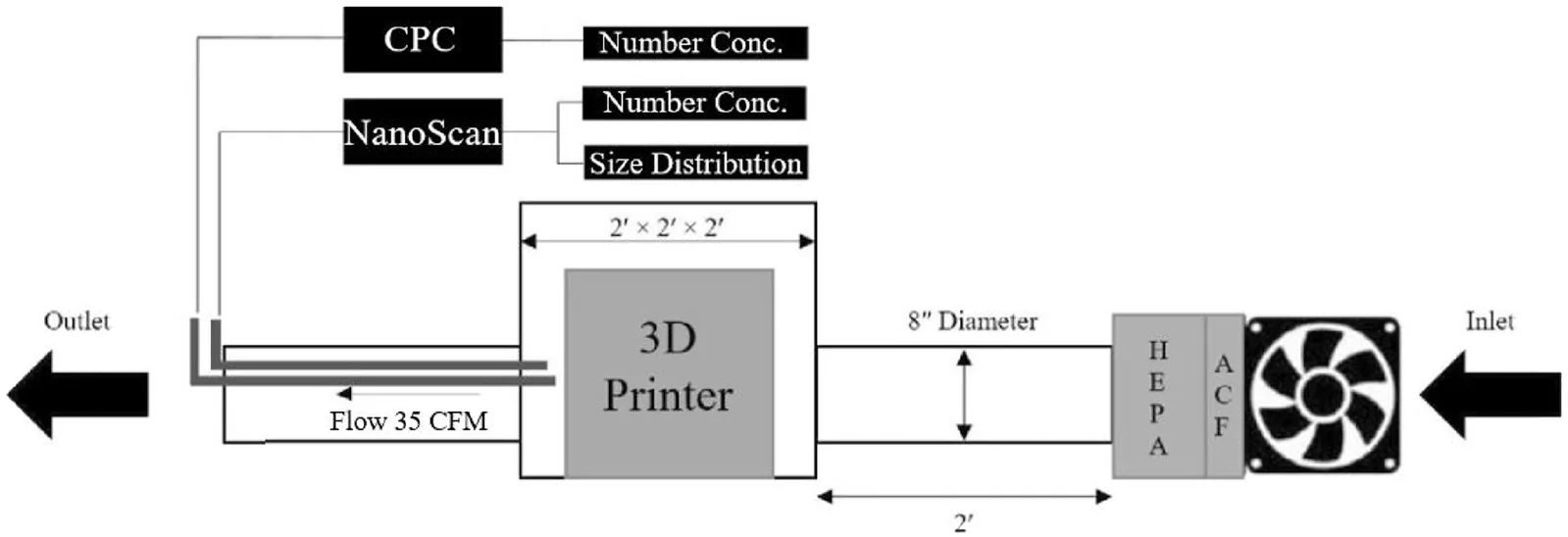
Printer emission test chamber schematic showing dimensions and sampling equipment. Schematic by NIOSH.

**Fig. 10.**
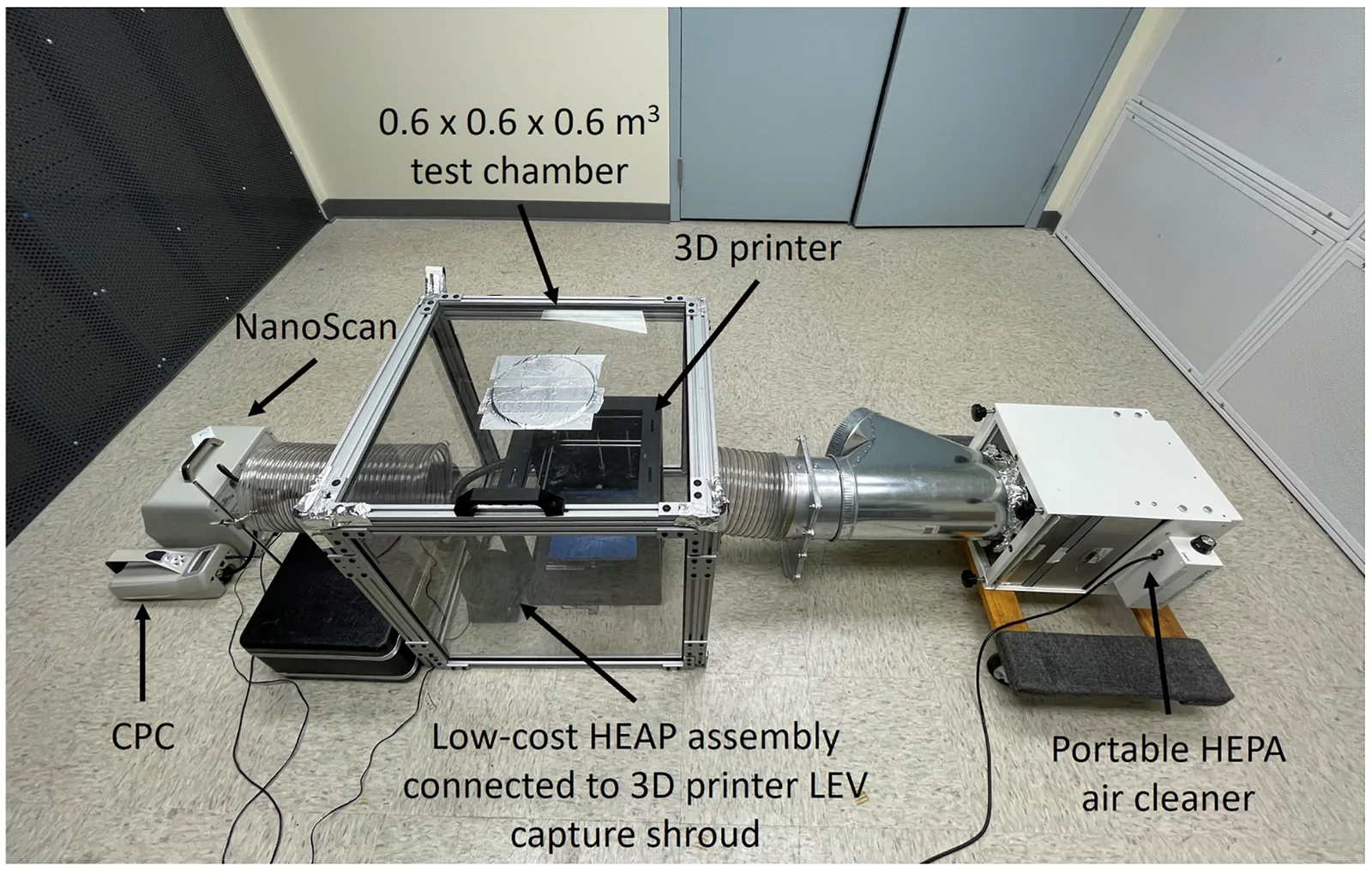
Actual printer emission test chamber with Printer 1 and sampling equipment. Photo by NIOSH.

**Fig. 11.**
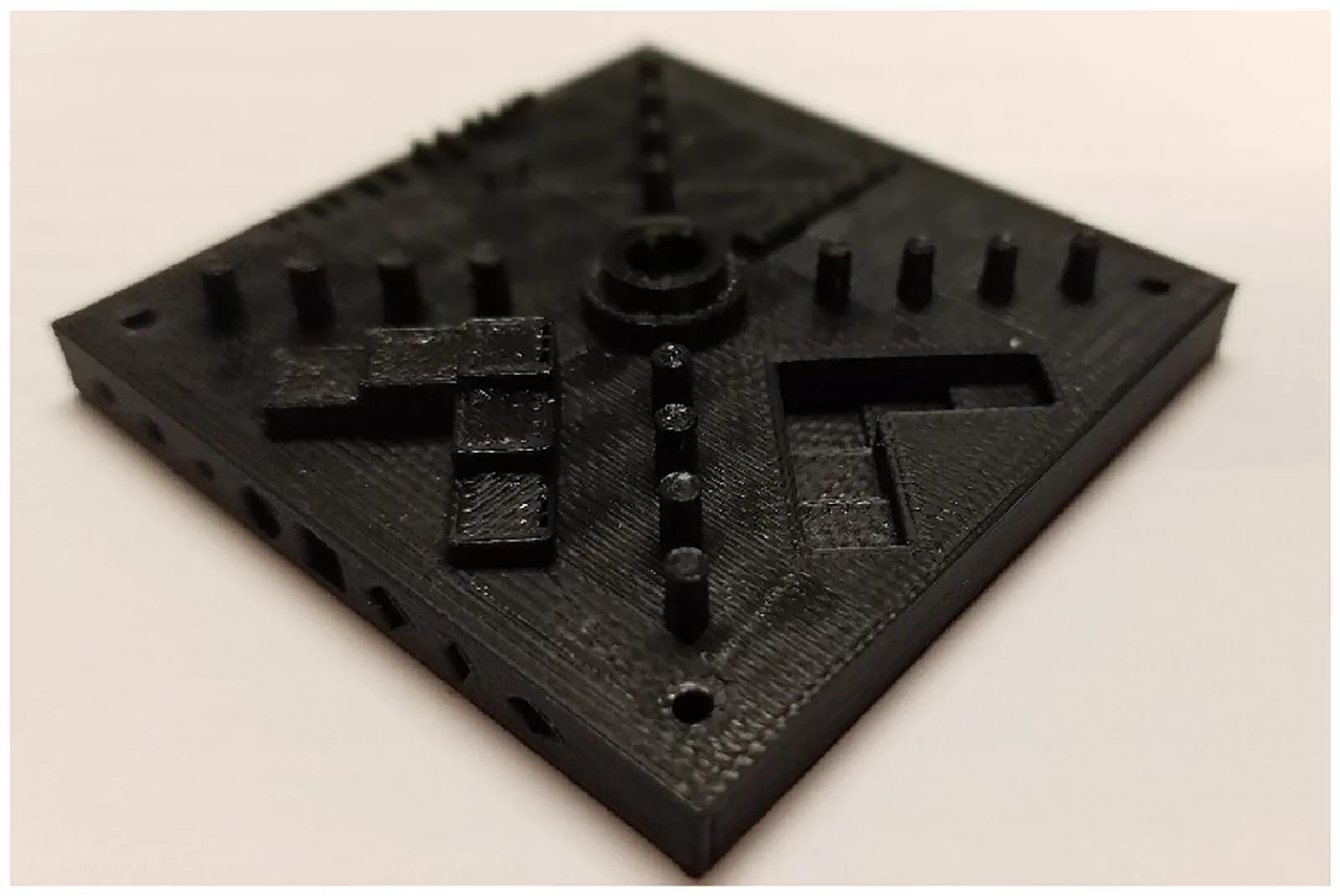
NIST Test Artifact printed in ABS. Photo by NIOSH.

**Table 1:** Description of local exhaust ventilation (LEV) Controls A – D

Control	Exhaust flow rate (m^3^/hr)	3D Printer used for Evaluation	Components
A	6.3	Printer 1	includes a custom 3D printed LEV extruder attachment and filter housing, HEPA filter^a^, 12 VDC blower fan, and flexible CPAP tubing with 22 mm cuffs.
B	2.4	Printer 2	
C	12.1	Printer 2	includes a commercially available, model-specific polycarbonate enclosure, custom 3D printer filter housing and exhaust duct, HEPA filter^a^, and 12 VDC blower fan.
D	21.2	Printer 2	includes a custom acrylic enclosure, 3D printed filter housing, high efficiency filter^b^, and 6.35 cm flexible tubing.

a high efficiency air vacuum filter with an H13 rating, composed of glass fiber media

b five-layer pleated paper shop vacuum filter with a 99% capture efficiency for particles 0.5 μm or larger

**Table 2.** Capture efficiency of Control A for particle emissions from Printer 1 during 3D printing test trials

Test #	No Control(#/cm^3^)	Control (#/cm^3^)	Capture Efficiency (%)
1	4408.53	1.46	99.97%
2	2242.81	0.86	99.96%
3	2811.58	0.48	99.98%
4	2255.02	0.34	99.98%
5	1743.29	0.75	99.96%
6	1903.79	16.22	99.15%
7	977.50	0.20	99.98%
8	2894.19	0.29	99.99%
9	331.85	0.54	99.85%
10	684.23	0.60	99.91%
**Average Capture Efficiency**			99.87%
**Test Statistic** *			99.72%

* Test Statistic = the minimum capture efficiency at the lower 95% confidence level

**Table 3.** Capture efficiency of Control B for particle emissions from Printer 2 during 3D printing test trials

Test #	No Control (#/cm^3^)	Control (#/cm^3^)	Capture Efficiency (%)
1	6719.40	41.75	99.38%
2	1177.04	10.83	99.08%
3	8102.86	20.10	99.75%
4	1153.55	17.47	98.49%
5	5175.72	810.51	84.34%
6	2770.26	124.77	95.50%
7	2672.49	19.20	99.28%
8	918.10	138.00	84.97%
9	2661.45	13.76	99.48%
10	85130.85	6502.37	92.36%
**Average Capture Efficiency**			95.26%
**Test Statistic**			91.75%

* Test Statistic = the minimum capture efficiency at the lower 95% confidence level

**Table 4.** Capture efficiency of Control C for particle emissions from Printer 2 during 3D printing test trials

Test #	No Control (#/cm^3^)	Control (#/cm^3^)	Capture Efficiency (%)
1	1325.83	1815.22	−36.91%
2	397.95	385.08	3.23%
3	1890.13	118.92	93.71%
4	3257.56	244.85	92.48%
5	1112.07	83.91	92.45%
6	610.49	33.98	94.43%
7	1029.63	1068.87	−3.81%
8	2618.49	755.16	71.16%
9	1659.27	291.33	82.44%
10	4061.10	619.84	84.74%
**Average Capture Efficiency**			57.39%
**Test Statistic**			28.55%

* Test Statistic = the minimum capture efficiency at the lower 95% confidence level

**Table 5.** Capture efficiency of Control D for particle emissions from Printer 2 during 3D printing test trials

Test #	No Control (#/cm^3^)	Control (#/cm^3^)	Capture Efficiency (%)
1	9826.75	20.23	99.79%
2	1828.36	1.86	99.90%
3	2729.94	4.21	99.85%
4	2504.90	3.50	99.86%
5	2063.54	4.53	99.78%
6	3776.48	4.30	99.89%
7	2810.41	8.95	99.68%
8	5443.93	7.30	99.87%
9	3353.74	4.41	99.87%
10	2683.52	6.93	99.74%
**Average Capture Efficiency**			99.82%
**Test Statistic**			99.78%

* Test Statistic = the minimum capture efficiency at the lower 95% confidence level

## Data Availability

Data will be made available upon request. It has also been included in supplementary material file.

## Appendix A. Supplementary data

Supplementary data to this article can be found online at https://doi.org/10.1016/j.aeaoa.2026.100432.
