# Supplementary material for: Design and evaluation of four low-cost engineering controls for reducing particle emissions from 3D printing
Source: Atmos Environ X. Author manuscript; Available in PMC 2026 Oct 24. (PMC13693809; doi:10.1016/j.aeaoa.2026.100432)
Supplement: Supplementary file 1 [file NIHMS2182942-supplement-Supplementary_Material.docx]

Supplemental Information

By Colleen O’Connor^a^, Christopher Barnes^a^, Kevin L. Dunn^a^, and Duane Hammond^a^

^a^Division of Field Studies and Engineering, National Institute for Occupational Safety and Health, Centers for Disease Control and Prevention, Cincinnati, OH 45226, USA

**Technical Specification of Fused Filament Fabrication 3D printers and engineering controls used in experiments**

**Table S1.** Technical specifications of 3D Printers 1 and 2

| **Printer** | **Build Volume (mm)** | **Filament Diameter (mm)** | **Nozzle Size (mm)** | **Max Print Speed (mm/s)** | **Max Nozzle Temp (°C)** | **Max Bed Temp (°C)** | **Cost (USD)** |
| --- | --- | --- | --- | --- | --- | --- | --- |
| 1 | 200 × 200 × 175 | 1.75 | 0.4 | 120 | 260 | 100 | $559 |
| 2 | 160 × 160 × 180 | 2.85 | 0.5 | 300 | 290 | 120 | $1,495 |

**Temporal behavior of particle emission with and without engineering controls in operations**

Fig. S1 – S4 illustrate particle number concentrations measured using a Nanoscan Scanning Mobility Particle Sizer (SMPS) (Model 3910, TSI Inc., Shoreview, MN, USA) during tests of Controls A – B in our emissions test chamber described in Section 2.2. These figures represent the highest minimum capture efficiencies observed across the ten tests performed on Controls A – B.

**Fig. S1.** Particle number concentrations measured by a Nanoscan Scanning Mobility Particle Sizer (SMPS) from Test 8 of evaluating Control A on Printer 1.

Control A Test 8

particle concentration (#/cm3)

| Time (min) | Test 8 Off |
| --- | --- |
| 1 | 0 |
| 2 | 1.8583 |
| 3 | 2.0926 |
| 4 | 1.1621 |
| 5 | 3.4793 |
| 6 | 1.6947 |
| 7 | 0 |
| 8 | 32.7505 |
| 9 | 55908 |
| 10 | 64439.3 |
| 11 | 17334.2 |
| 12 | 13977 |
| 13 | 9609.8 |
| 14 | 7786.82 |
| 15 | 7815.36 |
| 16 | 8375.71 |
| 17 | 4912.75 |
| 18 | 4140.96 |
| 19 | 2515.05 |
| 20 | 3188.34 |
| 21 | 2834.75 |
| 22 | 2119.84 |
| 23 | 1425.97 |
| 24 | 1350.18 |
| 25 | 1184.25 |
| 26 | 1022.06 |
| 27 | 1241.14 |
| 28 | 962.064 |
| 29 | 769.921 |
| 30 | 709.208 |
| 31 | 679.689 |
| 32 | 468.54 |
| 33 | 384.3 |
| 34 | 290.323 |
| 35 | 256.104 |
| 36 | 233.831 |
| 37 | 157.727 |
| 38 | 191.757 |
| 39 | 165.255 |
| 40 | 150.523 |
| 41 | 158.427 |
| 42 | 187.52 |
| 43 | 121.001 |
| 44 | 102.769 |
| 45 | 112.119 |
| 46 | 101.027 |
| 47 | 143.548 |
| 48 | 129.72 |
| 49 | 64.5847 |
| 50 | 34.769 |
| 51 | 79.4407 |
| 52 | 108.269 |
| 53 | 94.6516 |
| 54 | 139.206 |
| 55 | 51.4015 |
| 56 | 88.0076 |
| 57 | 132.64 |
| 58 | 89.4676 |
| 59 | 73.7885 |
| 60 | 92.4009 |
| 61 | 123.415 |
| 62 | 83.2201 |
| 63 | 70.22 |
| 64 | 91.7547 |
| 65 | 84.0122 |
| 66 | 118.951 |
| 67 | 91.79 |
| 68 | 67.3052 |
| 69 | 62.9017 |
| 70 | 58.0766 |
| 71 | 54.4984 |
| 72 | 60.7743 |
| 73 | 77.5798 |
| 74 | 161.883 |
| 75 | 3.4861 |
| 76 | 0 |
| 77 | 1.8845 |
| 78 | 0.5759 |
| 79 | 2.3242 |
| 80 | 1.0419 |
| 81 | 29.6083 |
| 82 | 0 |
| 83 | 2.995 |
| 84 | 0.9618 |
| 85 | 0 |
| 86 | 0.9422 |
| 87 | 0 |
| 88 | 0 |

|  | Test 8 On |
| --- | --- |
| 1 | 0 |
| 2 | 0 |
| 3 | 0 |
| 4 | 0.9422 |
| 5 | 1.1774 |
| 6 | 0 |
| 7 | 0 |
| 8 | 0 |
| 9 | 0 |
| 10 | 3.3848 |
| 11 | 2.2162 |
| 12 | 0 |
| 13 | 0 |
| 14 | 0 |
| 15 | 0 |
| 16 | 0 |
| 17 | 1.1774 |
| 18 | 0 |
| 19 | 0 |
| 20 | 0.6046 |
| 21 | 0 |
| 22 | 1.8138 |
| 23 | 0 |
| 24 | 0 |
| 25 | 0 |
| 26 | 0 |
| 27 | 0 |
| 28 | 0 |
| 29 | 0 |
| 30 | 0 |
| 31 | 0 |
| 32 | 0 |
| 33 | 0 |
| 34 | 0.6049 |
| 35 | 0 |
| 36 | 0 |
| 37 | 1.2168 |
| 38 | 0 |
| 39 | 0 |
| 40 | 0 |
| 41 | 0 |
| 42 | 0.6562 |
| 43 | 0 |
| 44 | 0 |
| 45 | 0 |
| 46 | 0 |
| 47 | 0 |
| 48 | 0.9425 |
| 49 | 0 |
| 50 | 0 |
| 51 | 0 |
| 52 | 0.5759 |
| 53 | 0 |
| 54 | 0 |
| 55 | 0 |
| 56 | 0 |
| 57 | 0 |
| 58 | 0 |
| 59 | 0 |
| 60 | 0 |
| 61 | 0 |
| 62 | 0 |
| 63 | 0 |
| 64 | 0 |
| 65 | 0 |
| 66 | 4.6484 |
| 67 | 1.1621 |
| 68 | 0 |
| 69 | 1.1621 |
| 70 | 0 |
| 71 | 0 |
| 72 | 0 |
| 73 | 0 |
| 74 | 0 |
| 75 | 0 |
| 76 | 0 |
| 77 | 0 |
| 78 | 0 |
| 79 | 0 |
| 80 | 0 |
| 81 | 0 |
| 82 | 0 |
| 83 | 0 |
| 84 | 0 |

**Note:** The primary y-axis on the left represents particle number concentrations without Control A in operation, while the secondary y-axis on the right represents particle number concentrations with Control A in operation.

**Fig. S2.** Particle number concentrations measured by a Nanoscan Scanning Mobility Particle Sizer (SMPS) from Test 3 of evaluating Control B on Printer 2.

Control B Test 3

particle concentration (#/cm3)

| Time (min) | Test 3 Off |
| --- | --- |
| 1 | 3.048 |
| 2 | 0 |
| 3 | 97.0436 |
| 4 | 64.622 |
| 5 | 70662.7 |
| 6 | 60195.4 |
| 7 | 60324 |
| 8 | 60139.7 |
| 9 | 31539.4 |
| 10 | 31783.9 |
| 11 | 33239.9 |
| 12 | 31119 |
| 13 | 26891.5 |
| 14 | 26319 |
| 15 | 22213.5 |
| 16 | 21295.2 |
| 17 | 19500.3 |
| 18 | 7693.2 |
| 19 | 4009.76 |
| 20 | 5398.82 |
| 21 | 3423.83 |
| 22 | 2677.88 |
| 23 | 1621.38 |
| 24 | 1155.1 |
| 25 | 1041.04 |
| 26 | 630 |
| 27 | 425.375 |
| 28 | 605.54 |
| 29 | 751.332 |
| 30 | 362.101 |
| 31 | 343.642 |
| 32 | 314.096 |
| 33 | 312.119 |
| 34 | 332.078 |
| 35 | 352.313 |
| 36 | 325.5 |
| 37 | 315.728 |
| 38 | 269.28 |
| 39 | 256.357 |
| 40 | 230.714 |
| 41 | 341.166 |
| 42 | 388.266 |
| 43 | 247.693 |
| 44 | 320.893 |
| 45 | 347.752 |
| 46 | 294.106 |
| 47 | 408.844 |
| 48 | 503.02 |
| 49 | 217.365 |
| 50 | 381.393 |
| 51 | 188.22 |
| 52 | 244.818 |
| 53 | 466.973 |
| 54 | 373.4 |
| 55 | 111.147 |
| 56 | 263.846 |
| 57 | 162.197 |
| 58 | 126.696 |
| 59 | 401.524 |
| 60 | 161.56 |
| 61 | 135.532 |
| 62 | 123.06 |
| 63 | 64.9323 |
| 64 | 75.6231 |
| 65 | 95.3805 |
| 66 | 108.022 |

|  | Test 3 On |
| --- | --- |
| 1 | 0.6451 |
| 2 | 0 |
| 3 | 0 |
| 4 | 976.342 |
| 5 | 12.0373 |
| 6 | 0.4387 |
| 7 | 0 |
| 8 | 0 |
| 9 | 0 |
| 10 | 0 |
| 11 | 0 |
| 12 | 0 |
| 13 | 0 |
| 14 | 0 |
| 15 | 1.4335 |
| 16 | 0.4178 |
| 17 | 0 |
| 18 | 0 |
| 19 | 0 |
| 20 | 0 |
| 21 | 0 |
| 22 | 0 |
| 23 | 0.6836 |
| 24 | 4.3382 |
| 25 | 1.2296 |
| 26 | 1.5876 |
| 27 | 0 |
| 28 | 1.3673 |
| 29 | 2.9748 |
| 30 | 1.7661 |
| 31 | 4.5741 |
| 32 | 5.3762 |
| 33 | 6.2958 |
| 34 | 5.2695 |
| 35 | 3.3078 |
| 36 | 3.3913 |
| 37 | 5.4477 |
| 38 | 5.3852 |
| 39 | 8.8624 |
| 40 | 15.9249 |
| 41 | 33.5891 |
| 42 | 16.8952 |
| 43 | 4.5513 |
| 44 | 21.4966 |
| 45 | 21.9796 |
| 46 | 14.0563 |
| 47 | 21.2621 |
| 48 | 11.3942 |
| 49 | 2.7379 |
| 50 | 8.8165 |
| 51 | 6.3675 |
| 52 | 6.4328 |
| 53 | 8.4484 |
| 54 | 2.6794 |
| 55 | 2.3466 |
| 56 | 3.8394 |
| 57 | 7.1169 |
| 58 | 8.7377 |
| 59 | 9.7966 |
| 60 | 4.8612 |
| 61 | 2.5697 |
| 62 | 51.7713 |
| 63 | 5.9751 |
| 64 | 0 |
| 65 | 0 |
| 66 | 0 |
| 67 | 0 |

**Note:** The primary y-axis on the left represents particle number concentrations without Control B in operation, while the secondary y-axis on the right represents particle number concentrations with Control B in operation.

**Fig. S3.** Particle number concentrations measured by a Nanoscan Scanning Mobility Particle Sizer (SMPS) from Test 6 of evaluating Control C on Printer 2.

Control C Test 6

particle concentration (#/cm3)

| Time (min) | Test 6 Off |
| --- | --- |
| 1 | 68.6532 |
| 2 | 4.0032 |
| 3 | 0.7703 |
| 4 | 18544.5 |
| 5 | 5286.63 |
| 6 | 2995.23 |
| 7 | 2274.09 |
| 8 | 2391.89 |
| 9 | 1512.77 |
| 10 | 1242.12 |
| 11 | 1356.18 |
| 12 | 947.412 |
| 13 | 1629.58 |
| 14 | 142.944 |
| 15 | 11.6338 |
| 16 | 0.9784 |
| 17 | 0.8698 |
| 18 | 0.9747 |
| 19 | 1.8037 |
| 20 | 0 |
| 21 | 0.7703 |
| 22 | 1.8176 |
| 23 | 1.253 |
| 24 | 2.2479 |
| 25 | 4.195 |
| 26 | 5.9657 |
| 27 | 3.1906 |
| 28 | 3.9617 |
| 29 | 2.8129 |
| 30 | 8.2978 |
| 31 | 9.3715 |
| 32 | 9.1121 |
| 33 | 7.8501 |
| 34 | 14.3508 |
| 35 | 11.8251 |
| 36 | 15.6048 |
| 37 | 13.7032 |
| 38 | 17.0573 |
| 39 | 19.1529 |
| 40 | 21.3022 |
| 41 | 32.6896 |
| 42 | 20.9997 |
| 43 | 32.2805 |
| 44 | 16.527 |
| 45 | 43.4181 |
| 46 | 31.9837 |
| 47 | 24.3668 |
| 48 | 33.5054 |
| 49 | 6.0266 |
| 50 | 3.4283 |
| 51 | 17.3743 |
| 52 | 17.8186 |
| 53 | 24.0068 |
| 54 | 5.4809 |
| 55 | 6.2888 |
| 56 | 8.0522 |
| 57 | 8.7594 |
| 58 | 10.6494 |
| 59 | 7.0652 |
| 60 | 3.789 |
| 61 | 5.4364 |
| 62 | 115.422 |
| 63 | 7.4219 |
| 64 | 1.4826 |

|  | Test 6 On |
| --- | --- |
| 1 | 55.5942 |
| 2 | 0.253 |
| 3 | 10.9449 |
| 4 | 1107.78 |
| 5 | 267.866 |
| 6 | 87.5056 |
| 7 | 62.8581 |
| 8 | 63.0591 |
| 9 | 150.272 |
| 10 | 79.0051 |
| 11 | 69.7407 |
| 12 | 47.7519 |
| 13 | 45.1431 |
| 14 | 2.338 |
| 15 | 2.8737 |
| 16 | 2.9003 |
| 17 | 0.447 |
| 18 | 0.9434 |
| 19 | 0 |
| 20 | 0 |
| 21 | 0.8705 |
| 22 | 1.4201 |
| 23 | 0.6966 |
| 24 | 0.447 |
| 25 | 0 |
| 26 | 0.8592 |
| 27 | 0.872 |
| 28 | 0.8993 |
| 29 | 0 |
| 30 | 0 |
| 31 | 0 |
| 32 | 0 |
| 33 | 0.8993 |
| 34 | 0 |
| 35 | 0 |
| 36 | 0 |
| 37 | 0 |
| 38 | 0 |
| 39 | 0 |
| 40 | 0 |
| 41 | 0 |
| 42 | 0.4258 |
| 43 | 0 |
| 44 | 0 |
| 45 | 0 |
| 46 | 1.9045 |
| 47 | 0 |
| 48 | 0.8705 |
| 49 | 0 |
| 50 | 0.447 |
| 51 | 0 |
| 52 | 0 |
| 53 | 0 |
| 54 | 0 |
| 55 | 1.5208 |
| 56 | 0 |
| 57 | 1.7411 |
| 58 | 0.9747 |
| 59 | 0.4258 |
| 60 | 0 |
| 61 | 0 |

**Note:** The primary y-axis on the left represents particle number concentrations without Control C in operation, while the secondary y-axis on the right represents particle number concentrations with Control C in operation.

**Fig. S4.** Particle number concentrations measured by a Nanoscan Scanning Mobility Particle Sizer (SMPS) from Test 2 of evaluating Control D on Printer 2.

Control D Test 2

particle concentration (#/cm3)

| Time (min) | Test 2 Off |
| --- | --- |
| 1 | 91.4326 |
| 2 | 2.5509 |
| 3 | 0.872 |
| 4 | 70732.1 |
| 5 | 27038.8 |
| 6 | 10349.1 |
| 7 | 6643.6 |
| 8 | 6300.27 |
| 9 | 4041.79 |
| 10 | 4224.1 |
| 11 | 2645.3 |
| 12 | 1798.18 |
| 13 | 1817.18 |
| 14 | 465.81 |
| 15 | 31.8632 |
| 16 | 5.4312 |
| 17 | 1.4173 |
| 18 | 1.4845 |
| 19 | 1.1717 |
| 20 | 1.5266 |
| 21 | 0.8592 |
| 22 | 0.8592 |
| 23 | 0 |
| 24 | 0 |
| 25 | 0.8368 |
| 26 | 1.62 |
| 27 | 4.0917 |
| 28 | 2.4313 |
| 29 | 4.6417 |
| 30 | 3.9806 |
| 31 | 9.0635 |
| 32 | 8.3574 |
| 33 | 17.9685 |
| 34 | 15.371 |
| 35 | 13.2348 |
| 36 | 23.5414 |
| 37 | 26.0494 |
| 38 | 24.9555 |
| 39 | 27.9141 |
| 40 | 33.4212 |
| 41 | 31.4014 |
| 42 | 23.4018 |
| 43 | 10.8233 |
| 44 | 32.7569 |
| 45 | 16.8554 |
| 46 | 19.2682 |
| 47 | 32.9329 |
| 48 | 11.8407 |
| 49 | 2.8192 |
| 50 | 3.3971 |
| 51 | 8.3054 |
| 52 | 11.6978 |
| 53 | 27.955 |
| 54 | 5.8766 |
| 55 | 4.166 |
| 56 | 10.4881 |
| 57 | 10.9365 |
| 58 | 12.9988 |
| 59 | 10.0688 |
| 60 | 6.6931 |
| 61 | 6.8137 |
| 62 | 5.271 |
| 63 | 386.999 |
| 64 | 15.3998 |
| 65 | 0.447 |
| 66 | 0.8705 |
| 67 | 0.894 |
| 68 | 0.447 |
| 69 | 0.7111 |
| 70 | 0.7703 |
| 71 | 0 |
| 72 | 0.8592 |
| 73 | 0 |
| 74 | 0 |
| 75 | 0 |

|  | Test 2 On |
| --- | --- |
| 1 | 6.1889 |
| 2 | 1.7411 |
| 3 | 0 |
| 4 | 0.7703 |
| 5 | 31.9853 |
| 6 | 10.5265 |
| 7 | 8.4056 |
| 8 | 2.3906 |
| 9 | 6.3197 |
| 10 | 8.4456 |
| 11 | 5.3659 |
| 12 | 4.9845 |
| 13 | 1.6688 |
| 14 | 1.2494 |
| 15 | 0.8592 |
| 16 | 1.6418 |
| 17 | 1.713 |
| 18 | 0 |
| 19 | 0.7703 |
| 20 | 0 |
| 21 | 0.7703 |
| 22 | 0.8993 |
| 23 | 1.7985 |
| 24 | 0.8993 |
| 25 | 0 |
| 26 | 0 |
| 27 | 0.7703 |
| 28 | 0 |
| 29 | 1.0394 |
| 30 | 0.8705 |
| 31 | 0 |
| 32 | 0.7703 |
| 33 | 0 |
| 34 | 0 |
| 35 | 0 |
| 36 | 0 |
| 37 | 0.8592 |
| 38 | 0 |
| 39 | 0 |
| 40 | 0 |
| 41 | 0.8993 |
| 42 | 0 |
| 43 | 0.8993 |
| 44 | 0.4463 |
| 45 | 0 |
| 46 | 0.8705 |
| 47 | 0 |
| 48 | 2.9262 |
| 49 | 0.8993 |
| 50 | 0.8705 |
| 51 | 2.2071 |
| 52 | 0 |
| 53 | 0 |
| 54 | 0 |
| 55 | 0 |
| 56 | 1.7184 |
| 57 | 0 |
| 58 | 0 |
| 59 | 0.9434 |
| 60 | 1.5208 |
| 61 | 1.3997 |
| 62 | 0.8592 |
| 63 | 1.3376 |
| 64 | 3.0318 |
| 65 | 0.8993 |
| 66 | 0.4258 |
| 67 | 0 |
| 68 | 0.8705 |

**Note:** The primary y-axis on the left represents particle number concentrations without Control D in operation, while the secondary y-axis on the right represents particle number concentrations with Control D in operation

**Particle Size Distribution of Printers 1 and 2**

The size distribution of particle emissions from Printers 1 and 2 (Fig. S5) were plotted by taking the average of three samples around the peak number concentration measured by a Nanoscan SMPS from each test trial (ten in total) in our emissions test chamber described in Section 2.2 without a control in operation. For both printers, the particle size distribution remained consistent across all control configurations whether not enclosed (Control A on Printer 1 and Control B on Printer 2) or enclosed (Controls C and D on Printer 2).

**Fig. S5.** Average number weighted particle size distribution of Printer 1 (A) and Printer 2 (B) measured by a Nanoscan SMPS.

**Data File**

Data file for all Capture Efficiency calculations for Controls A – D and plots representing particle emissions measured with a Nanoscan SMPS for all test trials in our emissions test chamber described in Section 2.2.
